# Effect of 24% EDTA root conditioning on the outcome of modified coronally advanced tunnel technique with subepithelial connective tissue graft for the treatment of multiple gingival recessions: a randomized clinical trial

**DOI:** 10.1007/s00784-021-04151-9

**Published:** 2021-08-24

**Authors:** Bartłomiej Górski, Marcin Szerszeń, Tomasz Kaczyński

**Affiliations:** 1 Department of Periodontology and Oral Mucosa Diseases, Medical University of Warsaw, Stanisława Binieckiego St 6, 02-097 Warsaw, Poland; 2 Department of Dental Prosthetics, Medical University of Warsaw, Stanisława Binieckiego St 6, 02-097 Warsaw, Poland

**Keywords:** Esthetics, Ethylenediaminetetraacetic acid (EDTA), Modified coronally advanced tunnel technique, Multiple gingival recessions, Subepithelial connective tissue graft

## Abstract

**Objectives:**

To investigate effects of root conditioning with 24% ethylenediaminetetraacetic acid (EDTA) on the 12-month outcomes after treatment of multiple gingival recessions (GR) with modified coronally advanced tunnel (MCAT) and subepithelial connective tissue graft (SCTG).

**Materials and methods:**

Twenty patients with 142 GR were treated (72 test sites: SCTG + EDTA and 70 control sites: SCTG). Average and complete root coverage (ARC, CRC), gain in keratinized tissue width (KTW), gain in gingival thickness (GT), root esthetic coverage score (RES), and patient-reported outcome measures (PROMs) were evaluated at 12 months post-operatively.

**Results:**

Differences between pre- and post-operative values were statistically significant only within but not between treatment modalities. At 12 months, ARC was 86.0% for SCTG + EDTA-treated and 84.6 for SCTG-treated defects (*p* = 0.6636). CRC was observed in 90.2% (tests) and 91.4% (controls) of all cases (*p* = 0.9903). Professional assessment of esthetic outcomes using RES showed highly positive results reaching the value of 8.9 in case of test sites and 8.7 for control sites (*p* = 0.3358). Severity of pain and swelling did not differ between sites, regardless of whether EDTA was used.

**Conclusions:**

Test and control sites presented similarly positive outcomes related to root coverage, periodontal and esthetic parameters, and patient satisfaction and self-reported morbidity with no statistical differences between them 12 months after surgery. No significant differences in evaluated variables were observed between sites treated with and without 24% EDTA.

Clinical relevance

Considering the limitations of the present study, the use of 24% EDTA for root conditioning did not improve 12-month outcomes after treatment of multiple RT1 and RT2 gingival recessions with MCAT and SCTG.

Trial registration

ClinicalTrials.gov identifier: NCT03354104

## Introduction

Gingival recession (GR) is an apical displacement of the gingival margin with the concomitant exposure of a portion of the root cementum. It is often associated with root caries, esthetic, and hypersensitivity concerns, which constitute significant therapeutic problems for patients. If left untreated, the progression of GR was estimated to be 0.4 mm over an average follow-up of 4 years [1]. GR affects population with both poor and high oral hygiene [2]. Quite recently, the prevalence of GR was reported in 91.6% of evaluated subjects and decreased to 70.7% when only the esthetic zone was considered [3].

A plethora of techniques and different flap designs have been developed for the surgical treatment of GR. Zabalegui et al. [4] described the tunnel approach that preserved the integrity of the papillae and did not require vertical releasing incisions when obtaining root coverage, which may increase coronal blood supply. Tunnel approach showed great potential in correcting GR [5]. Distinct modifications to the original tunnel technique have been described over time by several authors to improve clinical outcomes [6–8]. Although the efficacy of the tunnel technique was dependent on the application of subepithelial connective tissue graft (SCTG), which was regarded as the gold standard [9], attempts have been made to search for materials alternative to SCTG, such as collagen porcine dermal matrix [10], enamel matrix derivatives [11], and concentrated growth factors [12]. In order to achieve good esthetic results, the understanding how to apply scientific evidence for a given case is crucial [13]. Lately, Aroca et al. [14] proposed site-specific, selective use of SCTG in an attempt to reduce the amount of connective tissue harvested from the palate. In a recent systematic review and meta-analysis, the extreme efficacy of modified coronally advanced tunnel technique (MCAT) in treating multiple GR defects was confirmed [15]. Average root coverage (ARC) of 87.87% (± 16.45) and complete root coverage (CRC) of 57.46% were calculated. However, there has recently been an emphasis on alignment between professional (surrogate) end points and patient-centered outcomes (true end points) for the evaluation of root coverage procedures [16].

Exposed root surfaces can sometimes display a hypermineralized layer of cementum and endotoxin contamination; hence, mechanical and chemical preparation of the exposed root was pinpointed to influence the treatment outcome of root coverage procedures [17]. The aims of mechanical preparation were to remove demineralization areas and caries, to smooth out any irregularities, and to reduce pronounced root convexity [18]. On the other hand, the use of chemical agents has been suggested for decontaminating of the root surface area, removing the smear layer created by root instrumentation, and enhancing the attachment of the connective tissue by the exposure of collagen fibers and amount of patent dentinal tubules of the root surface [19, 20]. Different root modifiers have been proposed and the most commonly used chemical agents were the following: ctric acid, ethylenediaminetetraacetic acid (EDTA), tetracycline, doxycycline, fibronectin, and fibrin glues [21]. Owing to its quick and easy handling, EDTA became particularly popular among dental practitioners. Nevertheless, evidence supporting its clinical use in periodontal plastic surgery remains scarce and inconsistent [12, 22, 23]. Critical analysis of the available data constitutes the main tool in the decision-making process, but based on the available data, it is not possible to clearly elucidate about the use of EDTA in root coverage procedures [24].

Despite several clinical trials that tested the effectiveness and predictability of MCAT for the correction of multiple gingival recessions in distinct clinical settings, the effectiveness of 24% EDTA when combined with MCAT + SCTG has not yet been determined. Therefore, this randomized clinical trial aimed to study the impact of EDTA root conditioning on clinical outcomes after surgical treatment of multiple gingival recessions with MCAT + SCTG. Another goal was to assess root coverage esthetic score (RES) for professional esthetic evaluation and to analyze patient-reported outcome measures (PROMs).

## Materials and methods

### Study design

**Fig. 1:**
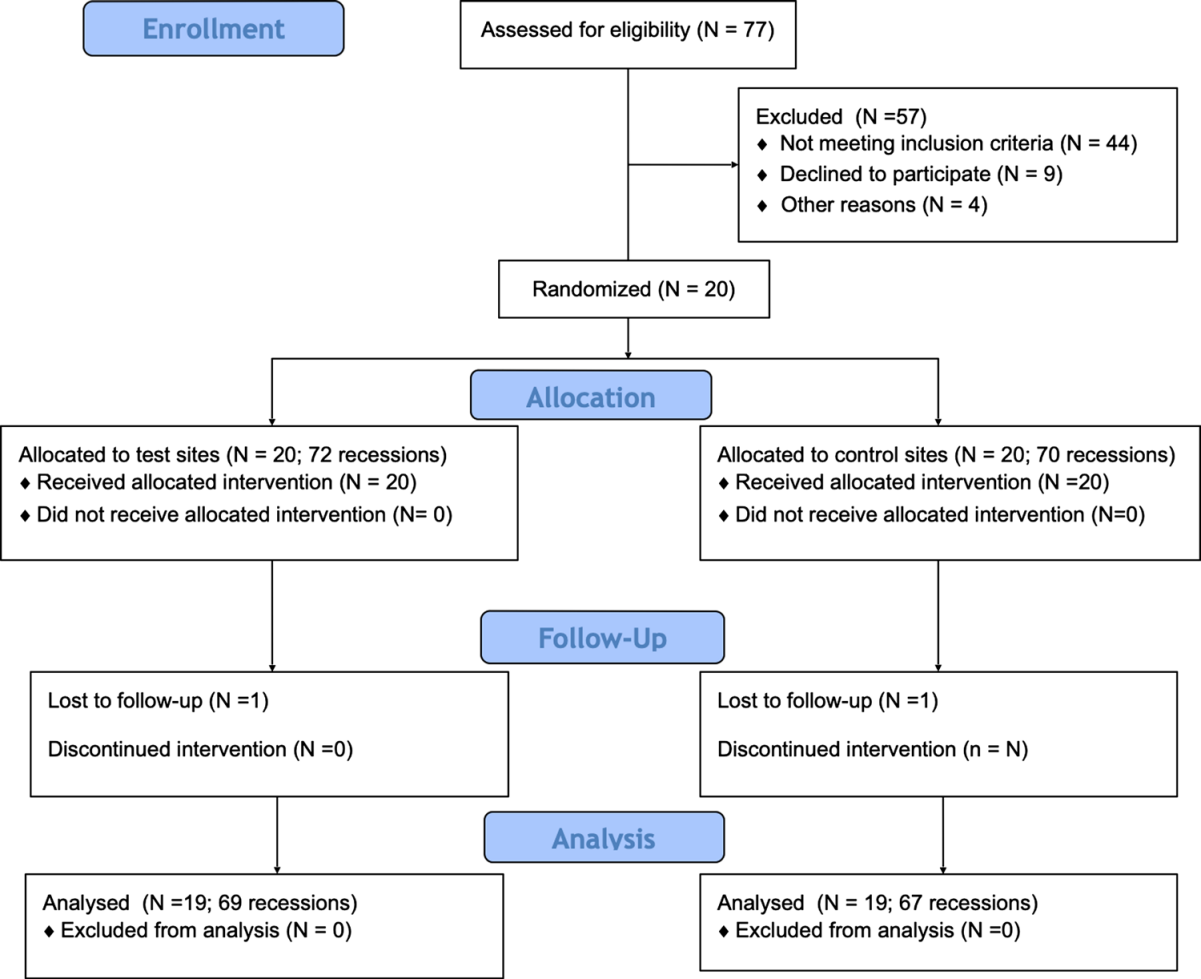
Consort diagram showing the study design

This study was a single-center, double-blinded, split-mouth, randomized controlled clinical trial including twenty patients (11 women and 9 men, aged 21–36; mean age 28.87 ± 4.46 years) who were recruited among patients referred to the Department of Periodontology and Oral Mucosa Diseases of Medical University of Warsaw between January 2019 and April 2020 (Fig. 1). The study was carried out in accordance with the Helsinki Declaration of 1975, as revised in Tokyo in 2004 after approval of the study design by the Bioethics Committee of Medical University of Warsaw (KB/208/2017; ClinicalTrials.gov ID: NCT03354104).

### Sample size calculation

Taking into account that percentage of root coverage was the primary objective and based on the data indicating that standard deviation (SD) of the differences in the paired measurements would not surpass 30%, the sample size for paired continuous data was calculated to be 18 subjects per treatment group [25]. This would provide 80% power to disclose a true difference of 20% points between test and control. However, considering that some patients might be lost during follow-up, 20 patients were recruited.

### Investigator calibration

Six non-study patients with at least two contralateral teeth with gingival recessions were enrolled for the calibration exercise. The designated examiner (DP) made clinical measurements twice at an interval of 24 h in 24 defects. Calibration was accepted when ≥ 90% of the recordings were repeated within a difference of 1.0 mm, and an exact agreement was reproduced in 75% of measurements.

### Subject population

The inclusion criteria were as follows: (1) presence of at least two adjacent gingival recessions type I (RT1: no loss of interproximal attachment) and/or II (RT2: the amount of interproximal attachment loss was less than buccal attachment loss) at least 1 mm deep on the buccal aspect of homologous maxillary or mandibular teeth, (2) full-mouth plaque score (FMPS) < 20%, (3) full-mouth bleeding on probing (FMBOP) < 20%, (5) presence of detectable cemento-enamel junction (CEJ), and (6) age ≥ 18 years. The following exclusion criteria were established: (1) active periodontal disease, (2) caries lesions or restorations in the cervical area, (3) systemic diseases with compromised healing potential or infectious diseases, (4) use of medications affecting periodontal status, (5) smoking, and (6) pregnancy or lactation.

One examiner (TK) qualified subjects into the study. Each patient signed an informed consent form before enrollment. Once the selected patients agreed to participate in the study, they were provided with detailed oral hygiene instructions on how to use the roll technique with a soft toothbrush. The full-mouth professional tooth cleaning was performed. Twenty patients were enrolled in the study, and one hundred forty-two gingival recessions were treated with MCAT in combination with SCTG either with (test, 72 defects) or without (control, 70 defects) 24% EDTA.

### Primary and secondary outcome variables

Percentages of root coverage and complete root coverage (ARC, CRC) at 12 months were the primary outcome variables. As secondary outcome variables, reduction in gingival recession height (GR), reduction in recession width (RW), gain in clinical attachment level (CAL), increase in gingival thickness (GT), increase in keratinized tissue width (KTW), root coverage esthetic score (RES) values, and patient-centered outcomes were established.

### Clinical measurements

Clinical parameters were evaluated for each gingival recession before the surgery under local anesthesia by a single-blind examiner (DP) with a graded periodontal probe (UNC probe 15 mm, Hu-Friedy, Chicago, IL, USA) and rounded off to the nearest 0.5 mm. The following parameters were recorded: (1) GR: distance from CEJ to the gingival margin at mid-buccal point of the tooth; (2) RW: horizontal distance measured between the mesial and distal margin of the recession at CEJ level; (3) probing pocket depth (PPD): distance from the gingival margin to the bottom of the gingival sulcus at mid-buccal point of the tooth; (4) clinical attachment level (CAL): distance from CEJ to the bottom of the gingival sulcus at mid-buccal point of the tooth; (5) KTW: distance between the gingival margin and the muco-gingival junction (MGJ) in the mid-buccal aspect of the tooth; (6) GT: measured at mid-buccal point of the tooth 3 mm apical to the gingival margin with the use of endodontic spreader 25 ISO (Poldent, Warsaw, Poland) and a silicon stopper positioned perpendicularly to the gingival surface until the alveolar bone or root surface was reached, an electronic caliper (YATO YT-7201, Toya, Wrocław, Poland), with 0.01-mm accuracy was selected to calculate GT value; (7) FMPS: the percentage of total surfaces (four aspects per tooth) that revealed the presence of plaque [26]; and (8) FMBOP: the percentage of total points (four points per tooth: mesio-buccal, mid-buccal, disto-buccal, mid-lingual) that bled after gentle probing [27].

At 12 months, GR, RW, PPD, CAL, KTW, and GT were again recorded by the same examiner and the percentage of root coverage was measured.

### Randomization and allocation concealment

Randomization had been carried out before surgical treatment by a statistician not involved in the study, using a computerized random number generator. Allocation of treatment sites was concealed in sealed and opaque envelopes, and revealed to the surgeon immediately before procedure. One envelope was opened to designate the surgical site located to the right to one of the two treatment modalities; consequently, the surgical site to the left was treated in accordance with the opposite treatment protocol. No information on treatment allocation was given to the patient.

### Surgical treatment

All surgical procedures were performed by the same surgeon (BG) in line with the modified coronally advanced tunnel technique [6]. Both sides were treated during the same appointment (the right side was always treated first). After local anesthesia with 4% articaine hydrochloride with adrenaline (1:100,000) (Ubistesin Forte 1.7 ml, 3-M ESPE, Saint Paul, MN, USA), the surgical area was prepared as a full-thickness flap up to MGJ and as a split-thickness flap beyond MGJ. The papillary regions were detached in their buccal aspects with the periosteum. The exposed root surfaces were planed using designated curettes (Gracey Curettes, Hu-Friedy). In the next step, SCTG was harvested from the palate using the de-epithelialized graft technique [28]. After removing epithelium, the thickness of SCTG was less than 1 mm, its width was around 4 mm, and its length corresponded with the length of the recipient area. Donor site was covered by a hemostatic sponge which was stabilized with cross-mattress non-resorbable sutures (Seralon 4/0 18 mm 3/8, Serag-Wiessner GmbH & Co. KG, Neila, Germany). In the next step, in case of the test site, the root surfaces were conditioned with 24% EDTA (PrefGel, Straumann, Basel, Switzerland) for 2 min and washed with saline. SCTG was placed into the tunnel and stabilized at CEJ level with resorbable sling sutures (PGA Resorba 6/0 11 mm 3/8, RESORBA Medical GmBH, Nürnberg, Germany). Subsequently, the buccal flap was advanced coronally to fully cover SCTG and secured with 6/0 non-resorbable monofilament sling sutures (Seralon 6/0 12 mm 3/8, Serag-Wiessner GmbH & Co). On the control site, the recipient area was prepared in the same manner, but 24% EDTA was not used.

### Post-operative instructions and patient-reported evaluation of morbidity

After the surgery, patients received 400 mg of ibuprofen and were asked to take the second dose 8 h later, as well as any additional tablets later on if required. It was suggested that they avoid brushing, flossing, and chewing in the treated area for 2 weeks and rinse the mouth twice daily for 1 min using 0.2% chlorhexidine solution. Furthermore, patients were provided with a self-report questionnaire to evaluate pain and swelling using visual analog scale (VAS). Each VAS consisted of a horizontal line, 10 cm (100 mm) in length, with a statement at each end representing a single extreme of the assessed variable (the scale was anchored by “no pain or swelling” as score 0 and “worst imaginable pain or swelling” as score 100). The questionnaires were self-completed on the 1st, 2nd, 4th, 7th, and 14th days after surgery. Check-up appointments were scheduled for 1, 2, and 4 weeks and later at 3, 6, and 12 months, during which plaque control was provided. The sutures were removed 14 days after the surgery and patients were instructed in mechanical tooth cleaning of the operated sides using a soft toothbrush and the roll technique.

### Professional esthetic evaluation and patient-reported satisfaction

The esthetic outcome was assessed post-operatively after 12 months by an independent second examiner (MS), who was blinded to the treatment assignment, in accordance with RES [29]. The evaluation was based on comparing digital photographs taken at baseline and after 12 months. Five variables were assessed: (1) the level of gingival margin (GM); (2) marginal tissue contour (MTC); (3) soft tissue texture (STT); (4) muco-gingival junction alignment (MGJ); and (5) gingival color (GC). A score of 0, 3, or 6 was used for evaluation of GM, whereas a score of 0–1 was used for each of the other variables. The ideal esthetic score was 10.

At the 12-month follow-up examination, questionnaires were given to the patients for subjective evaluation of esthetics and overall satisfaction. Questions were designed in a dichotomous fashion (yes or no), and VAS was used to evaluate esthetic satisfaction.

### Statistical analysis

Descriptive statistics were performed using mean values, standard deviations (SD), frequencies, and percentages. Normality of distribution for quantitative variables was assessed using the Shapiro–Wilk test. All quantitative variables were normally distributed. Therefore, the Student *t* test was used to compare means between two treatment groups. Comparison of fractions (percentages) was carried out using Pearson’s chi-square test. The following calculations were made: (1) recession reduction = GR0 − GR12, (2) ARC = GR0 − GR12/GR0 × 100%, (3) CAL gain = CAL0 − CAL 12, (4) KTW gain = KTW12 − KTW0, and (5) GT gain = GT12 − GT0. The two-way ANOVA was used to determine the difference between treatment groups for patients’ VAS-reported pain and swelling on the 1st, 2nd, 4th, and 7th days after the surgery. The analyses were performed using the R 3.2.3 software (R Core Team 2019) and statistical significance was established for *p* < 0.05.

## Results

### Patient and defect characteristics

A total of 142 gingival recessions were treated (72 defects in the SCTG + EDTA group and 70 defects in the SCTG group). Study teeth were maxillary incisors (30), canines (27), premolars (48), and first molars (10), as well as mandibular canines (6), premolars (17), and first molars (4). Fifteen subjects had recessions in the maxillary arch, and other five showed defects in the mandibular arch. The majority of treated teeth were upper premolars (Table 1). Baseline data was homogeneous and well-balanced for all of the 20 involved subjects (Table 2).

**Table 1:** Characteristics for test and control groups

Variables	Baseline		12 months	
	SCTG + EDTA (*N* = 20; *n* = 72)	SCTG (*N* = 20; *n* = 70)	SCTG + EDTA (*N* = 19; *n* = 69)	SCTG (*N* = 19; *n* = 67)
Tooth type (*n*)				
Incisors	15	15	15	15
Canines	17	16	17	16
Premolars	33	32	31	29
Molars	7	7	6	6
Tooth position (*n*)				
Maxillary teeth	57	58	57	55
Mandibular teeth	15	12	12	12
Type of GR according to Cairo (*n*, %)				
RT1	49 (68.06)	47 (67.14)	-	-
RT2	23 (31.94)	23 (32.86)	-	-

*SCTG* subepithelial connective tissue graft,* N* number of patients, *n* number of defects, *GR* gingival recession, *RT* recession type

**Table 2:** Clinical parameters (mean and standard deviation) at baseline and 12 months after surgery

	Baseline	12 months	*p*
GR SCTG + EDTA (mm)	1.80 (1.26)	0.26 (0.72)	< 0.0001*
GR SCTG	1.55 (1.34)	0.23 (0.59)	< 0.0001*
*p*	0.1376	0.7166	
ARC SCTG + EDTA (%)		86.08 (31.76)	
ARC SCTG		84.69 (30.82)	
*p*		0.6636	
CRC SCTG + EDTA (%)		65 (90.28)	
CRC SCTG		64 (91.43)	
*p*		0.9903	
GR red SCTG + EDTA (mm)		1.56 (1.18)	
GR red SCTG		1.51 (1.35)	
*p*		0.7547	
RW SCTG + EDTA (mm)	2.76 (1.92)	0.53 (1.30)	< 0.0001*
RW SCTG	2.36 (2.05)	0.53 (1.29)	< 0.0001*
*p*	0.1264	0.9785	
PPD SCTG + EDTA (mm)	1.42 (0.52)	1.58 (0.62)	0.0110
PPD SCTG	1.46 (0.57)	1.58 (0.70)	0.0093*
*p*	0.5683	0.9248	
CAL SCTG + EDTA (mm)	2.73 (1.55)	1.34 (1.10)	< 0.0001*
CAL SCTG	2.45 (1.67)	1.27 (1.14)	< 0.0001*
*p*	0.1798	0.6711	
CAL gain SCTG + EDTA (mm)		1.34 (1.10)	
CAL gain SCTG		1.27 (1.14)	
*p*		0.6711	
KTW SCTG + EDTA (mm)	3.14 (1.64)	3.86 (1.28)	0.0021*
KTW SCTG	3.00 (1.36)	3.76 (1.33)	< 0.0001*
*p*	0.4727	0.6000	
KTW gain SCTG + EDTA (mm)		0.78 (1.18)	
KTW gain SCTG		0.79 (1.00)	
*p*		0.2690	
GT SCTG + EDTA (mm)	1.33 (0.47)	1.93 (0.63)	< 0.0001*
GT SCTG	1.25 (0.33)	1.81 (0.52)	< 0.0001*
*p*	0.0545	0.3166	
GT gain SCTG + EDTA (mm)		0.61 (0.51)	
GT gain SCTG		0.52 (0.49)	
*p*		0.3166	

*GR* gingival recession height, *SCTG* subepithelial connective tissue graft, *ARC* average root coverage, *CRC* complete root coverage, *GR red* gingival recession reduction, *RW* gingival recession width, *PPD* probing pocket depth, *CAL* clinical attachment level, *KTW* keratinized tissue width, *GT* gingival thickness. *Statistically significant (*p* ≤ 0.05)

Healing was uneventful in all patients. One patient was lost before the 12-month follow-up. Consequently, a total of 136 defects were analyzed in 19 subjects 1 year after surgical treatment. The study design and flow are shown in Fig. 1.

### Clinical outcomes

Descriptive statistics for the clinical parameters evaluated at baseline and 12 months after surgery are presented in Table 2. At 12 months, PPD values were not statistically different within and between groups. Significant improvements in GR, RW, and CAL were observed in both groups 12 months post-operatively compared with the baseline measurements, but no statistically significant differences between treatment groups were seen. In the test group, the mean recession height decreased significantly from 1.8 ± 1.2 (baseline) to 0.2 ± 0.7 mm (12 months), with a percentage of ARC of 86 ± 31 and a CRC in 65 out of 72 (90.2%) recession defects. In the control group, mean recession height decreased significantly from 1.0 ± 1.3 to 0.2 ± 0.5 mm, with a percentage of ARC of 84 ± 30 and a CRC in 64 out of 70 (91.4%) recession defects. There was also a statistically significant CAL gain in both groups (1.3 ± 1.1 and 1.2 ± 1.1 mm for the test and control groups, respectively). Two treatment modalities allowed for a significant gain in KTW and GT on both sites: in case of KTW, from 3.1 ± 1.6 to 3.8 ± 1.2 mm on the SCTG + EDTA site and from 3.0 ± 1.3 to 3.7 ± 1.3 mm on the SCTG site; in case of GT, from 1.3 ± 0.4 to 1.9 ± 0.6 on the SCTG + EDTA site and from 1.2 ± 0.3 to 1.8 ± 0.5 mm on the SCTG site. No significant differences with respect to CAL gain, WKT gain, and GT gain at 12 months between two study groups were found.

**Fig. 2:**
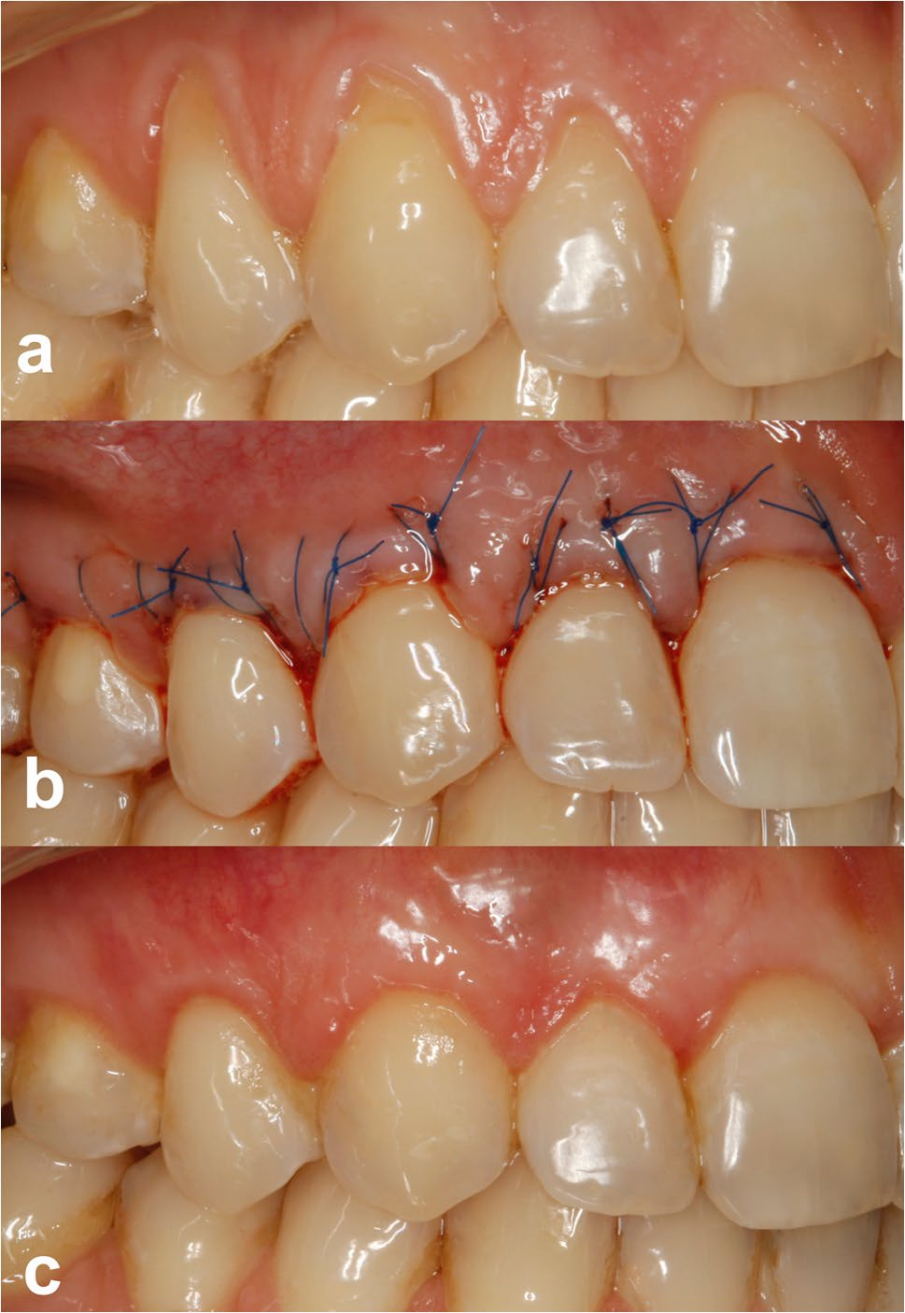
**a** Pre-operative view of gingival recessions on test side. **b** Immediate post-operative view. **c** 12-month post-operative view

**Fig. 3:**
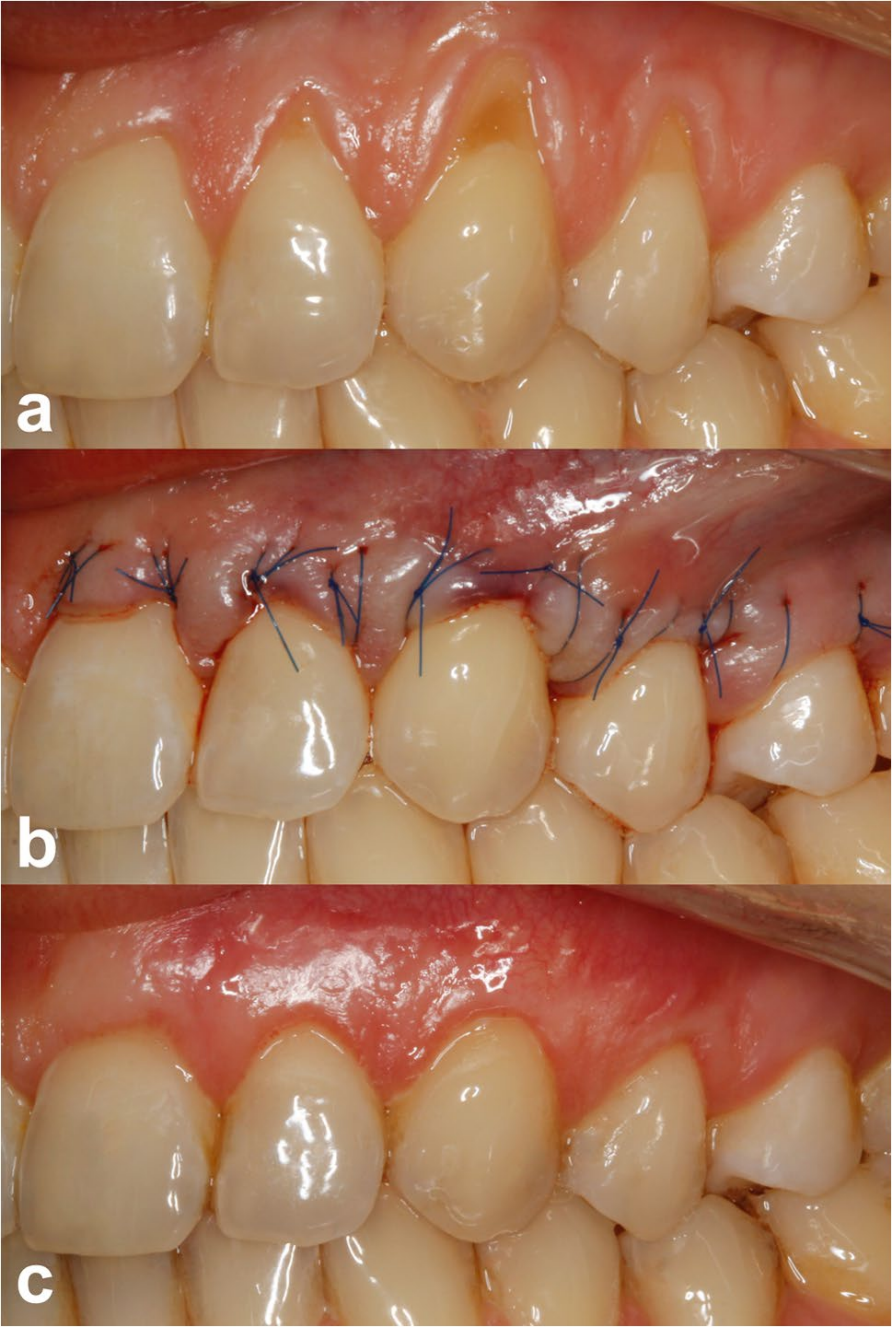
**a** Pre-operative view of gingival recessions on control side. **b** Immediate post-operative view. **c** 12-month post-operative view

Clinical outcomes of one of the patients are shown in Fig. 2 and Fig. 3.

### Professional esthetic evaluation

Both treatments showed high esthetic results. The root coverage esthetic score in the SCTG + EDTA group was 8.9 ± 1.3, whereas in the SCTG group 8.7 ± 1.3 (*p* = 0.3358) (Table 3). No statistically significant differences in assessed parameters of RES between the two treatment modalities were found. Keloid formation was not observed in any patient after 12 months.

**Table 3:** Evaluation of esthetic outcomes after 12 months (mean and standard deviation)

	GM	MTC	STT	MGJ	GC	RES
SCTG + EDTA	5.51 (1.12)	0.87 (0.34)	0.86 (0.35)	0.88 (0.33)	0.91 (0.28)	8.98 (1.30)
SCTG	5.47 (1.15)	0.80 (0.40)	0.83 (0.38)	0.86 (0.35)	0.87 (0.34)	8.79 (1.31)
*p*	0.8447	0.2234	0.5464	0.6636	0.3461	0.3358

*SCTG* subepithelial connective tissue graft, *GM* gingival margin, *MTC* marginal tissue contour, *STT* soft tissue texture, *MGJ* muco-gingival junction alignment, *GC* gingival color, *RES* root coverage esthetic score

### Patient-reported outcome measures

The values on the post-operative VAS are presented in Table 4. Slight pain was reported on 14 (70%) test sites and on 17 (85%) control sites. Greater pain intensity was reported on the 1st and 2nd days after surgery. By day 14, no pain was recorded for any of the patients. Slight to moderate swelling occurred in 18 (90%) subjects at day 1 post-surgery, and in 7 (35%) patients at day 7. By day 14, no edema was reported for any of the patients. There were no statistically significant differences in terms of pain or edema experience between two sites.

**Table 4:** Subject experience in term of post-operative morbidity

		SCTG + EDTA (*n* = 72)					SCTG (*n* = 70)					*p*
		1^st^ day	2^nd^ day	4^th^ day	7^th^ day	14^th^ day	1^st^ day	2^nd^ day	4^th^ day	7^th^ day	14^th^ day	
Pain	*N* answering “Yes” (%)	14 (70.00)	17 (85.00)	11 (55.00)	6 (30.00)	0 (0.00)	15 (75.00)	17 (85.00)	10 (50.00)	7 (35.00)	0 (0.00)	0.7691
	VAS mean (SD)	23.25 (23.41)	28.75 (22.18)	15.25 (18.32)	4.50 (7.59)	0 (0.00)	23.75 (24.91)	26.25 (21.21)	15.25 (19.49)	4.75 (7.15)	0.0 (0.00)	0.5392
Edema	*N *answering "Yes" (%)	20 (100.00)	20 (100.00)	20 (100.00)	6 (30.00)	0 (0.00)	19 (95.00)	20 (100.00)	19 (95.00)	7 (35.00)	0 (0.00)	0.9996
	VAS (SD)	46.75 (25.15)	48.50 (22.01)	29.00 (12.52)	3.25 (5.68)	0 (0.00)	47.50 (24.68)	48.75 (22.41)	31.00 (19.57)	5.25 (8.50)	0.00 (0.00)	0.8886

*SCTG* subepithelial connective tissue graft, *n*, *N* number, *VAS* visual analog scale, *SD* standard deviation. *Statistically significant (*p* ≤ 0.05)

Table 5 depicts the evaluation of esthetics and overall satisfaction by patients. No significant difference was detected between both sites with respect to gingival color, gingival contour, and gingival recession coverage, as measured by VAS values. When comparing treatment modalities, VAS assessments for overall patient satisfaction were generally high with nearly identical mean values of 78.9 ± 23.1 for the test site and of 84.7 ± 13.9 for the control site. Almost all patients declared a willingness to participate in the treatment again, and would recommend it to others.

**Table 5:** Results of patient questionnaire for evaluation of esthetics and overall satisfaction

Question	SCTG + EDTA (*n* = 72)		SCTG (*n* = 70)		*p*
	*N* answering “Yes” (%)	VAS mean (SD)	*N* answering “Yes” (%)	VAS mean (SD)	
Gingival color	20 (100%)	83.23 (13.35)	20 (100%)	82.11 (12.09)	0.8536
Gingival contour	19 (95%)	81.89 (14.90)	19 (95%)	81.17 (13.43)	0.9999
Recession coverage	19 (95%)	75.01 (19.57)	19 (95%)	79.44 (14.83)	0.4079
“How satisfied are you with the results of the surgery?”	18 (90%)	78.89 (23.11)	19 (95%)	84.69 (13.89)	0.9982
“Would you decide again to go for the treatment performed?”	18 (90%)	81.33 (16.61)	18 (90%)	81.94 (16.59)	0.9091
“Would you recommend the treatment to another person?”	18 (90%)	77.49 (16.74)	19 (95%)	83.84 (14.67)	0.9982

*SCTG* subepithelial connective tissue graft, *n*, *N* number, *VAS* visual analog scale, *SD* standard deviation. *Statistically significant (*p* ≤ 0.05)

## Discussion

The effect of EDTA root surface conditioning to improve outcomes of root coverage of multiple RT1 and RT2 gingival recessions with MCAT + SCTG remains unknown. To the best of our knowledge, this is the first study that evaluates the efficacy of 24% EDTA root treatment when the surgery is performed with modified coronally advanced tunnel and subepithelial connective tissue graft. Being as it may, the present investigation may add relevant information to the existing literature. At 12 months, ARC was 86.0% for SCTG-EDTA-treated defects and 84.6% for SCTG-treated defects. CRC was found in 90.2% (tests) and 91.4% (controls) of the cases. KTW gain was 0.78 mm on sites where roots were conditioned with EDTA, and 0.79 mm on sites without EDTA, whereas GT gain reached 0.61 and 0.52, respectively. However, complete root coverage is not the sole goal of treatment and final soft tissue quality, such as gingival margin contour, color, and texture, and lack of scar tissue formation are of utmost importance when root coverage procedures are evaluated. In the present study, the professional esthetic assessment carried out using RES revealed very high scores in terms of both tests (8.9) and controls (8.7). In this respect, reported outcomes are in agreement with those published in previous studies, where MCAT was applied [7, 30–32], and remain consistent with the conclusion of a recent systematic reviews and meta-analyses [15, 33]. Be that as it may, test and control sites presented similar outcomes related to root coverage and periodontal and esthetic parameters, with no statistical differences between them. Unfortunately, it is impossible to make direct comparisons with other studies due to lack of trials that would adapt a similar approach. Therefore, based on the findings presented in the current research, root surface modification with 24% EDTA provided no additional benefit 12 months after root coverage with MCAT + SCTG in terms of clinical and esthetic parameters. However, the question remains whether EDTA application may influence the stability of reported outcomes of surgical treatment in the longer term.

The healing of SCTG to the root surface demonstrated that the connection between the graft and the root was largely consisted of a combination of long junctional epithelium and connective tissue attachment, with fibers parallel to the root surface [34]. The use of root modifiers has been proposed by some authors in an attempt to enhance the success rate of root coverage by means of favoring attachment of the regenerated periodontal structures to the root surface. For instance, enamel matrix derivative showed the potential to promote substantial formation of the periodontal attachment apparatus on tooth roots with new bone, cementum, and periodontal ligament, as well as new attachment formation (connective tissue adhesion) [35, 36]. In this matter, more stable attachment showed less recession rebound and better coverage after 2 years [37]. By the same token, EDTA was found to remove the smear layer, expose collagen fibers, and enhance early cell colonization [38]. After conditioning with EDTA, root surface became a more biocompatible environment for attachment, growth, migration, proliferation, and differentiation of periodontal ligament cells under in vitro conditions [39]. In another in vitro study, EDTA alone or in combination with enamel matrix protein promoted enlargement, proliferation, and density of fibroblast [40]. It was also assumed that root conditioning might stabilize the bond between the fibrin of the blood clot and the root surface in the early healing process [41]. A clinical repair with fiber attachment would provide preferable functional permanence compared with long junctional epithelium [42]. Furthermore, it would grant stability of clinical attachment gain in the long term and boost reconstructive treatment goals. However, due to ethical concerns, it is impossible to evaluate the histological healing pattern between the root surface and SCTG and to determine the type of attachment achieved in clinical settings.

Very recent systematic review and meta-analysis revealed that adjunct application of EDTA may provide benefits when performing root coverage treatment with coronally advanced flap (CAF) + SCTG [22]. The authors reported statistically significant differences for GR reduction (3.68 mm versus 3.07 mm), CAL gain (4.15 mm versus 3.07 mm), and PD changes (− 0.44 mm versus 0.27 mm) in favor of the EDTA group. The outcomes of CRC and KTW gain were not significantly different. A possible explanation might be attributed to the several advantages of the CAF approach, especially increased access to root surface area in case of vertical releasing incision preparation, which also facilitates periosteal dissection [43]. These results are in contrast with our study, in which root conditioning with 24% EDTA did not seem to influence 12-month clinical outcomes since the abovementioned differences were not statistically significant between tests and controls. Reasons for this are open to speculation, but it might be associated with different surgical approaches used. One reasonable hypothesis may be related to the flap design. Without vertical releasing incisions and under application of a split-full-split flap preparation in MCAT, the flap is not fully detached from the underlying bone, papillae maintain their integrity, and root surfaces are not easily accessible, which is why the probability of contamination of root surface with blood or saliva is higher. Consequently, this may alter the effect of root modification and conditioning with 24% EDTA and decrease its potential accordingly. Similar conclusions on feasible limitations of flap design in root conditioning were drawn by Stähli et al. [31] who investigated the beneficial influence of enamel matrix derivative on clinical results using enamel matrix derivatives following treatment of single and multiple GR by the MCAT + SCTG. Quite interestingly, in another recent study, root conditioning before root coverage with a partial-thickness double-pedicle graft did not significantly affect the outcome [23].

With respect to patient-reported outcomes regarding early post-operative morbidity, as well as long-term esthetics and satisfaction, little research has been carried out on MCAT and none of hitherto existing studies evaluated the impact of additional root conditioning with 24% EDTA on this matter. In the present study, pain and swelling were reported by the majority of patients 1 day after the surgery and gradually decreased during the first week. The maximum severity of pain and edema was reported on the second day. No major adverse events were reported or observed. Mean intensity of pain described was low, whereas swelling intensity could be categorized as low or moderate. Two days after the surgery, mean VAS score was 28.7 in terms of pain and 48.5 in terms of swelling for the SCTG + EDTA sites, and 26.2 and 48.7 for the SCTG sites, respectively. No statistically significant differences were observed in the severity of early post-operative pain and swelling between sites treated with or without EDTA. It can be hypothesized that the extension and split preparation of flap itself may have negatively contributed to the patients’ early post-operative perception. Moreover, recent review and network meta-analysis concluded that SCTG-based techniques significantly increase patient morbidity compared to flap alone [33]. The present results compare well with those of previous studies that interpreted early post-operative patient-reported discomfort following treatment of GR with MCAT [31, 32]. With reference to the patients’ esthetic satisfaction, 12-month VAS value evaluated in the questionnaires for the SCTG + EDTA group was 78.9, whereas for the SCTG group 84.7 (*p* = 0.9982). Quite similarly, equally favorable results were also reported for both treatment modalities in terms of subjective perception of gingival color and marginal contour. All in all, the overall patient esthetic satisfaction was high. A total of 18 patients stated that they were willing to undergo further periodontal surgery if required. Generally speaking, 24% EDTA root conditioning prior to root coverage procedures with MCAT did not significantly affect patient-reported outcomes in the current study. The findings of our research are consistent with results of our previous study [32] and are slightly inferior to the outcomes presented by Zuhr et al. [44], who reported VAS values of 92.1 for tunnel-treated GR. One reason for discrepancy could be the fact that in the aforementioned study both single and multiple recessions were treated.

The current study has some limitations that should be addressed. The first is relatively short time-period of assessment, as the evaluation of clinical parameters should be performed in the medium to long term [45]. The second is the lack of histological evaluation of healing pattern; however, this was impossible for ethical reasons. Therefore, no comments could be made on the type of attachment and tissues formed. Last but not least, it should also be stated that results obtained in terms of GR coverage were affected by the strict inclusion criteria and by careful patient selection. The outcomes may have been different if the entry criteria had been modified. Furthermore, in vivo studies and prospective randomized studies with larger sample size and longer follow-ups are required to investigate the influence of root conditioning with 24% EDTA in the healing process after root coverage procedures, and to evaluate the long-term stability of clinical outcomes. Different types of gingival recessions defects in all types of teeth should be thoroughly investigated in future studies.

## Conclusions

Considering the limitations of the present study, it may be concluded that.

- MCAT + SCTG is an extremely effective plastic approach in treating multiple GR defects,
- The use of 24% EDTA for root conditioning did not affect 12-month clinical and esthetic outcomes in GR treatment with MCAT and SCTG,
- Patient-reported outcomes were not related to root surface conditioning with 24% EDTA,
- We have not found any evidence to support root conditioning with 24% EDTA prior to root coverage of multiple RT1 and RT2 gingival recessions with MCAT + SCTG.
